# Fibula osteal flap with proximal peroneal perforator skin paddle for composite oromandibular reconstruction: A case report

**DOI:** 10.1097/MD.0000000000023590

**Published:** 2020-12-11

**Authors:** Kang Liu, Wei Zhang, Yue Wang, Dan-Wei Xiang, Hai-Bo Shi, Qi-Lin Liu

**Affiliations:** a Department of Oral and Maxillofacial Surgery, School and Hospital of Stomatology, Jilin University, Changchun; b Department of Oral and Maxillofacial Surgery, Yantai Affiliated Hospital of Binzhou Medical University, Yantai, PR China.

**Keywords:** fibular flap, mandibular reconstruction, proximal perforator, skin paddle

## Abstract

**Rationale:**

Cutaneous perforators of peroneal vessels are divided into proximal and distal perforators on the basis of perforator distributions and musculocutaneous or septocutaneous properties. The traditional fibular osteocutaneous free flap is raised over the distal two-thirds of the fibula with a skin paddle based on distal perforators, which is affixed to the posterior crural septum. However, the skin pedicle may not be available due to anatomic variations or intraoperative injuries. Herein, because of the absence of distal perforators, we reserved and expropriated proximal perforators originating from the musculocutaneous branch of the superior part of the peroneal artery before it divided into nutrient and arcuate arteries and successfully harvested a separate osteal fibula and proximal perforator skin paddle with a single vascular pedicle-peroneal vessel.

**Patient concerns:**

A 62-year-old man with a 6-month history of mandibular swelling and soft tissue invasion was referred to us.

**Diagnosis:**

Panoramic radiography and computed tomography showed an irregular radiolucent lesion of the mandibular body, and histopathological analysis confirmed a follicular-pattern ameloblastoma.

**Interventions:**

The diseased mandible and soft tissue were resected and reconstructed with a vascularized fibular osteal flap with the proximal perforator skin paddle.

**Outcomes:**

The mandibular contour was successfully restored; the skin paddle in the mouth was in good condition after 8 months of follow-up.

**Lessons:**

The proximal perforator is reliable and practical for supplying a skin paddle and has significant potential for future applications. We recommend reserving the proximal perforator skin paddle as a backup flap when planning to raise a fibula flap, since unavailability or injury of the traditional fibular skin island based on distal perforators occurs frequently. This approach can avoid the exploration for a second donor site, save surgical time, and reduce surgical complexity. Moreover, we anticipate more frequent use of the proximal perforator flap in the future because of its flexibility and large volume, and since it can be combined with the osteal fibula or fibular osteocutaneous flap. However, an understanding of the traits of the proximal perforator and determination of its peroneal origin by computed tomography angiography is crucial for predesigning fibular osteal flaps with a proximal perforator skin paddle.

## 1 Introduction

Advancements in microsurgery and the development of various types of vascularized free flaps have transformed the field of head and neck reconstruction. While fibular free flaps, iliac crest free flaps, and scapular free flaps are the 3 workhorse flaps for composite hard and soft tissue reconstruction, the vascularized free osteocutaneous fibular flap is the preferred choice because of its unique advantages such as the availability of a long bone graft, the provision to allow 2-team working, and the presence of a long pedicle.

In 1975, the first successful transfer of a fibular graft without a skin paddle was performed in the reconstruction of a tibial defect.^[1]^ Subsequently, the osteocutaneous fibular flap was first reported in 1983 by Chen and Yan,^[2]^ who harvested the skin paddle based on perforators taken off from peroneal vessels to the skin. Since then, the anatomical characteristics and identification strategies for cutaneous branches of the peroneal artery have been extensively studied. After the first lower jaw reconstruction was performed using a fibular free flap in 1989 by Hidalgo,^[3]^ these flaps have become increasingly popular in oromandibular area reconstruction. Moreover, advancements have also been achieved in the raising technique.

Anatomically, the fibular flap's dominant vascular pedicle is the peroneal artery, which is derived from the tibial-fibular trunk. Together with the anterior and posterior tibial arteries, the peroneal artery represents 1 of the 3 main branches of the popliteal artery. Detailed anatomic studies and live surgery of the infrapopliteal vasculature have led to increased reliability of diversity in the cutaneous skin island.^[4,5]^

The perforators derived from the peroneal artery supply perfusion to the cutaneous portion of the flap, and in combination with the fibular graft, allow favorable reconstruction of composite soft- and hard-tissue defects. In a prospective study, Yu et al observed 2 distinct groups of perforators along the long axis of the fibula bone based on their distribution and named these perforators the proximal and distal perforators.^[5]^ Clinically the skin paddles are usually raised based on the perforators located rather distally. For complex defects such as a through-and-through defect, a second skin paddle can be designed based on the proximal perforator on the basis of the traditional fibular flap.

Undoubtedly, the precise design of the skin paddle of the fibular flap relies on precise mapping of perforator locations, sizes, and types. Here, we report an unusual case without distal perforators. On the basis of computed tomography angiography (CTA) findings, we harvested a separate osteal fibula and a skin paddle sharing single peroneal vessels, in which the skin paddle was supplied by the musculocutaneous branch of the proximal part of peroneal artery before it divided into nutrient and arcuate arteries. This new peroneal system free flap was successfully used to reconstruct composite defects of the mandible and soft tissue defects. Its future applications can be of great significance. Since the proximal perforator is not the preferred perforator, there are no reports describing the use of the osteal fibula with a single skin paddle based on a proximal perforator.

## 2 Case presentation

This study was approved by the ethics institutional review board of the hospital of stomatology, Jilin University.

**Figure 1:**
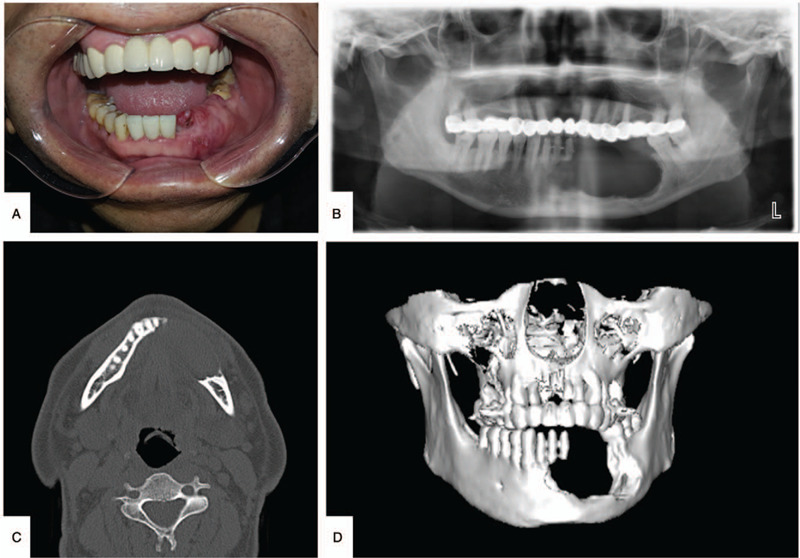
(A) Preoperative view of the intraoral mucosa of the patient with ameloblastoma; the surface of the oral mucosa was ruptured. Preoperative panoramic radiograph (B), horizontal CT scan (C), and 3-dimensional CT scan (D) showed an irregular radiolucent lesion of the mandibular body, exceeding the midline. CT = computed tomography.

**Figure 2:**
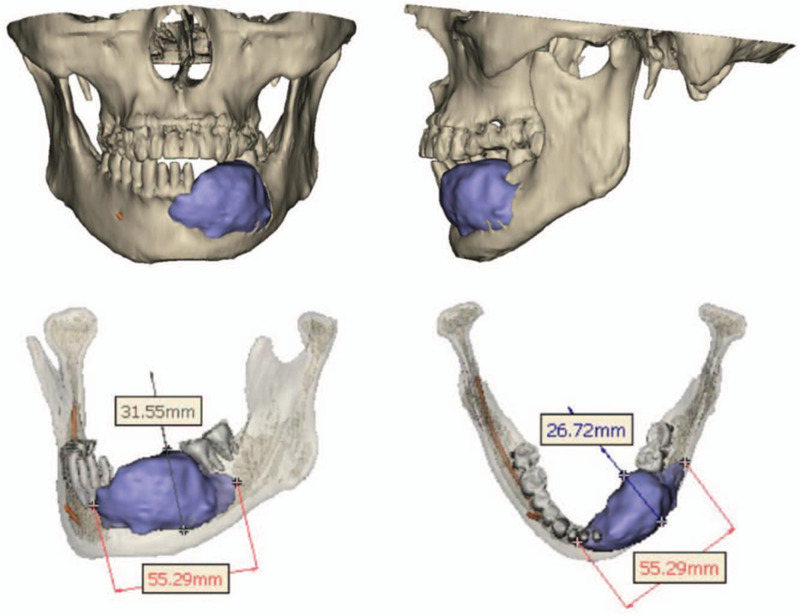
Preoperative 3-dimensional segmentation and reconstruction showed extension of the lesion.

A 62-year-old man was referred to us due to a 6-month history of mandibular swelling and soft tissue involvement. Intraoral examination revealed lingual and labial expansion of the left mandibular alveolar process along with a partially abnormal mucosa. Radiographic evaluation, including panoramic radiography and computed tomography, showed an irregular radiolucent lesion of the mandibular body (Fig. 1). Preoperative 3-dimensional segmentation and reconstruction showed that the tumor was about 55.3 mm ∗ 31.6  mm ∗ 26.7 mm (Fig. 2). Histopathological analysis confirmed a follicular-pattern ameloblastoma.

We planned to perform a segmental mandibulectomy with safety margins and simultaneous reconstruction with a free osteocutaneous fibular flap.

### 2.1 Perforator mapping

**Figure 3:**
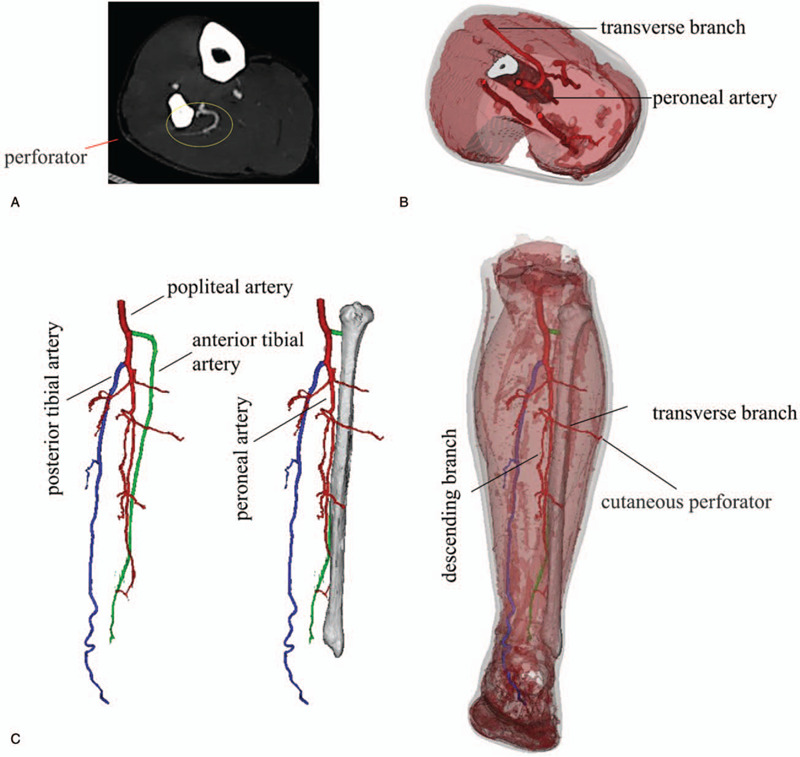
(A) Preoperative MIP image of CTA (Axial View) showed a proximal perforator (yellow circles) derived from the peroneal artery. (B) Three-dimension reconstruction CTA (Axial View) revealed the transverse branch course of the proximal perforator. (C) Three-dimension reconstruction CTA (Coronal View) revealed the transverse and descending branch course of the proximal perforator. To facilitate the observation of the peroneal artery, the colors of the anterior tibial artery and the posterior tibial artery were intentionally changed to green and blue, respectively. CTA = computed tomography angiography, MIP = maximum intensity projection.

Preoperative CTA was performed to confirm the existence of the anterior tibial, posterior tibial, and peroneal artery vasculature (Fig. 3). The cutaneous perforators at the lower shank were found and marked using Doppler ultrasonography.

RadiAnt Digital Imaging and Communications in Medicine Viewer (Medixant, Poznan, Poland) was used to reconstruct maximum intensity projection images. Mimics Medical 20.0 (Materialise, Leuven, Belgium) was used to convert CTA Digital Imaging and Communications in Medicine data into 3D objects. The distal perforators were not found, while the origin of the cutaneous perforators from the proximal peroneal artery was confirmed by vascular reconstruction. The discrepancy between CTA and Doppler mapping was noted, and a backup plan was made. Written informed consent was obtained from the patient preoperatively after he was given a detailed explanation of the operative procedures and backup planning.

### 2.2 Flap elevation

**Figure 4:**
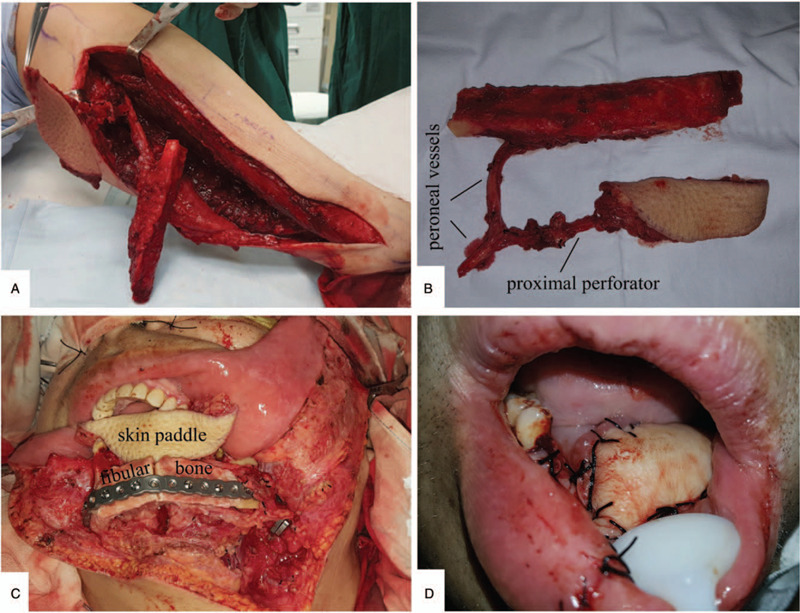
(A) During the fibular flap-raising process, the skin flap based on proximal perforator was harvested. (B) The fibular flap with a separate skin paddle had a common peroneal trunk. We shaped the flap in order to achieve the best match and contour possible; the fibular bone was used to reconstruct the mandible (C), and the skin flap was used to restore the soft tissue (D).

Under general anesthesia, the patient was placed in the supine position. The diseased mandible and soft tissues were resected. Simultaneously, the skin paddle was designed according to the perforators of the peroneal artery guided by Doppler ultrasonography, and an anterior approach was made via an incision along the anterior edge of the flap and dissected under a subfascial plane. We found that ultrasonic mapping had erroneously located the perforators, and we also confirmed the congenital absence of the distal perforators. By further exploring upstream based on CTA findings, a proximal musculocutaneous perforator was found and was traced to its origin from the peroneal artery. The skin paddle was then redesigned and the perforator was dissected retrogradely through the soleus to the source vessels similar to the process while raising a perforator skin flap. Finally, a fibular osteal flap with a separate skin paddle sharing a common peroneal trunk was harvested (Fig. 4A and B), and the composite defects were successfully reconstructed (Fig. 4C and D). The operation lasted for 7 hours, with an estimated blood loss of 300 mL.

### 2.3 Outcomes and follow-up

**Figure 5:**
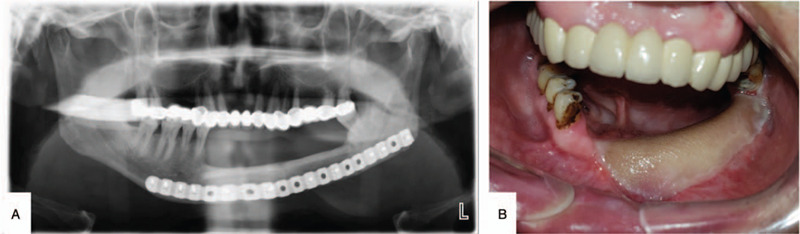
At 8 mo postoperatively, the follow-up panoramic radiograph indicated good bone healing. (A) The graft bone had incorporated well with the residual mandible, and the mandible contour was restored well. (B) The skin paddle in the mouth was in good condition and functioned well.

The patient was successfully cured without infection postoperatively and was satisfied with the result of reconstruction. Panoramic radiographs showed that the bone seams could be hardly observed at 8 months postoperation. The skin paddle in the mouth was also in good condition (Fig. 5). There was no recurrence of the tumor during the 2-year follow-up assessment.

## 3 Discussion

In the fibular osteocutaneous free flap, the nourishing vessels of the skin paddle are the cutaneous perforators from the peroneal artery. Because of anatomic variations or surgical injuries of the perforating vessels, many skin paddles cannot be successfully harvested.^[6,7]^ In such situations, clinicians have to take remedial measures. While small soft-tissue defects can be sutured directly with feeble tethering, the purely osseous flap can only be used for bony defect reconstruction without an inspection window to monitor the blood supply of the flap. However, in cases with large soft-tissue defects, to prevent extremely tight closure of the soft tissue that will lead to dehiscence and infection, the contralateral shank will have to be explored or another cutaneous flap will have to be selected to restore the soft tissue defect, which can increase morbidity of second donor-site and can also be time-consuming. In the present case, we raised a skin paddle based on the proximal peroneal artery, which successfully rescued the distal perforator absence and avoided exploration for a second donor site, thereby saving time and precluding the need for another set of recipient vessels.

The choice of the proximal peroneal perforator as a backup was based on our understanding of the features of the proximal perforator as well as CTA mapping data confirming its peroneal origin. Although the perforator anatomy has been deeply investigated and the concept of proximal and distal group perforators has been proposed, the proximal perforator skin paddle is not routinely included in the primary skin paddle in clinical practice.^[5,7–9]^ Cadaver dissections and clinical practice have demonstrated that the majority (70%–96%) of distal perforators are septocutaneous,^[5,6,10,11]^ while almost all proximal perforators are musculocutaneous,^[5]^ Surgeons use different approaches for designing the skin paddle according to the localization and understanding of perforators. Yu et al stated that the distal group of perforators, usually 1 to 3 in number, was generally located over the lower third quarter of the fibula, and was critical to the skin paddle design.^[5]^ In contrast, the proximal group of perforators was seldom used unless a 2–skin-island fibular flap is desired.^[5]^ It is less useful because of its very proximal location^[5]^ and also because of its musculocutaneous property. In this regard, the concept of predesigning the proximal perforator as a substitute will be significant, since skin paddle loss or unavailability may occasionally occur due to distal perforator loss or absence.

Anatomically, the skin paddle of the traditional osteocutaneous fibula flap is connected with bone by the posterior intermuscular septum. However, due to its unyielding nature, if the defect is complex, the skin paddle will be difficult to inset because of the limitation of movement between the bone and skin paddle.^[12,13]^ In some conditions, when the defect is extensive, this skin paddle also fails to offer enough soft-tissue volume.^[14]^ In contrast, the proximal perforator skin paddle is more flexible than the traditional free osteocutaneous fibular flap. Moreover, this skin paddle provides extended soft-tissue coverage and allows primary closure of the donor site.^[5]^

The flap used in our case was flexible since its structure was similar to that of a scapular free flap nourished by the circumflex scapular vascular system. However, the significant advantages of the proximal perforator skin paddle fibula free flap in comparison with the scapula osteocutaneous free flap are apparent: the fibula is a bicortical bone that is comparatively hard, thus suitable for weight-bearing early. The scapula on the other hand is an irregular bone, rich in cancellous bone. A sufficient bone length of the fibula can offer an approximately 26-cm bone graft in an adult, whereas the bone stock of the scapula may sometimes be insufficient. The fibula is also easier for bone shaping due to the arcuate artery property, while the scapula is not. However, the main disadvantage of scapular free-flap raising is the fact that the 2-team approach is sometimes not possible, and intraoperative position changes are time-consuming. Even though 2-team work can be performed by placing the patients in a tilted decubital position,^[15]^ flap raising is still cumbersome because of the proximity between the 2 operation teams. In contrast, fibular flap raising allows a 2-team approach by default.

In summary, our preliminary experience showed that the proximal perforator skin paddle fibular free flap is reliable and practical for the reconstruction of composite oromandibular defects. Since the proximal perforator skin paddle can be employed as a backup skin island when the distal peroneal perforator is absent or lost, in addition to the fact that the proximal perforator skin paddle fibular free flap offers the combined advantages of the traditional fibular osteocutaneous free flap and scapular osteocutaneous free flap, fibular flaps are expected to be used more frequently in clinical practice. Nevertheless, if a proximal perforator is taken into consideration, CTA mapping is recommended to confirm the skin location and peroneal origin of the proximal perforator.

## Author contributions

**Conceptualization:** Kang Liu, Wei Zhang and Qi-Lin Liu.

**Data curation:** Kang Liu, Yue Wang, Dan-Wei Xiang and Hai-Bo Shi.

**Formal analysis:** Kang Liu and Wei Zhang.

**Investigation:** Wei Zhang, Yue Wang and Qi-Lin Liu.

**Methodology:** Kang Liu, Wei Zhang and Qi-Lin Liu.

**Project administration:** Wei Zhang and Qi-Lin Liu.

**Resources:** Wei Zhang and Qi-Lin Liu

**Software:** Kang Liu, Yue Wang, Dan-Wei Xiang, Hai-Bo Shi and Qi-Lin Liu.

**Supervision:** Qi-Lin Liu.

**Writing – original draft:** Kang Liu.

**Writing – review & editing:** Wei Zhang, Hai-Bo Shi and Qi-Lin Liu.
